# An Audit and Comparison of pH, Measured Concentration, and Particulate Matter in Mannitol and Hypertonic Saline Solutions

**DOI:** 10.3389/fneur.2021.667842

**Published:** 2021-05-17

**Authors:** Christopher J. Carr, Jonathan Scoville, James Ruble, Chad Condie, Gary Davis, Candace L. Floyd, Logan Kelly, Ken Monson, Ethan Reichert, Buse Sarigul, Gregory W. J. Hawryluk

**Affiliations:** ^1^Department of Neurosurgery, Tulane University/Ochsner Clinic Foundation, New Orleans, LA, United States; ^2^Department of Neurosurgery, University of Utah School of Medicine, Salt Lake City, UT, United States; ^3^Department of Pharmacotherapy, University of Utah School of Medicine, Salt Lake City, UT, United States; ^4^Department of Physical Medicine and Rehabilitation, University of Utah School of Medicine, Salt Lake City, UT, United States; ^5^Department of Neurosurgery, Okmeydani Education Hospital, Istanbul, Turkey; ^6^Section of Neurosurgery, Department of Surgery, University of Manitoba, Health Sciences Centre, Winnipeg, MB, Canada; ^7^Brain Trauma Foundation, Palo Alto, CA, United States

**Keywords:** hyperosmolar therapy, audit, acid-base imbalance, pH, hypertonic saline, intracranial hypertension, mannitol, particulate matter

## Abstract

**Background:** The preferred hyperosmolar therapy remains controversial. Differences in physical properties such as pH and osmolality may be important considerations in hyperosmolar agent selection. We aimed to characterize important physical properties of commercially available hyperosmolar solutions.

**Methods:** We measured pH and concentration in 37 commonly-used hyperosmolar solutions, including 20 and 25% mannitol and 3, 5, 14.6, and 23.4% hypertonic saline. pH was determined digitally and with litmus paper. Concentration was determined by freezing point and vapor pressure osmometry. Salinity/specific gravity was measured with portable refractometry. Particulate matter was analyzed with filtration and light microscopy and with dynamic light scattering nephelometry.

**Results:** pH of all solutions was below physiological range (measured range 4.13–6.80); there was no correlation between pH and solution concentration (*R*^2^ = 0.005, *p* = 0.60). Mannitol (mean 5.65, sd 0.94) was less acidic than hypertonic saline (5.16, 0.60). 14/59 (24%) pH measurements and 85/111 concentration measurements were outside manufacturer standards. All 36/36 mannitol concentration measurements were outside standards vs. 48/72 (67%) hypertonic saline (*p* < 0.0001). All solutions examined on light microscopy contained crystalline and/or non-crystalline particulate matter up to several hundred microns in diameter. From nephelometry, particulate matter was detected in 20/22 (91%) solutions.

**Conclusion:** We present a novel characterization of mannitol and hypertonic saline. Further research should be undertaken, including research examining development of acidosis following hyperosmolar therapy, the relevance of our findings for dose-response, and the clinical relevance of particulate matter in solution.

## Introduction

Hyperosmolar solutions are critical therapeutics in modern neurocritical care, and they have a long history of use. In 1919, changes were demonstrated in cat brain volume following intravenous administration of hyperosmolar and hypoosmolar solutions (1). The following year, the use of hypertonic saline to decrease brain edema caused by tumors was reported (2). In the 1950s, urea became the first agent in widespread use for reducing intracranial pressure (3). Mannitol has long been a workhorse treatment for intracranial hypertension and was the recommended agent in the Brain Trauma Foundation's (BTF's) original *Guidelines for the Management of Severe Head Injury*, published in 1996 (4). In the current, 4th edition guidelines, mannitol remains the sole agent recommended; nevertheless, the BTF judged there to be insufficient evidence to support the superiority of any specific hyperosmolar agent (5–7). In contrast, the more recent *Guidelines for the Acute Treatment of Cerebral Edema in Neurocritical Care Patients* from the Neurocritical Care Society acknowledge recently published evidence in favor of hypertonic saline (8). Despite this new evidence, however, there remains uncertainty as to which agent is preferable overall and whether one agent may be preferred in specific clinical circumstances (8–30).

Despite the critical role of hyperosmolar therapy, we are unaware of any prior effort to systematically measure the physical properties of commercially available mannitol and hypertonic saline solutions that can be administered to patients. Differences in physical properties such as pH and concentration may be important considerations in selecting a specific agent, and a better understanding of precisely what is administered is anticipated to inform patient care. Per manufacturer specifications, a remarkably wide range of physical properties is permissible: for instance, pH may range from 4.5 to 7.0; measured solution concentration may contain labeled concentration ±5%. Given this, we were interested in auditing how variable measured properties of these solutions actually are and how these change with labeled concentration. We were also interested in determining whether solutions contain particulate matter given the potential for mannitol and hypertonic saline to crystalize. Finally, we were interested in determining whether generalizable differences in these properties exist between various labeled concentrations of mannitol and hypertonic saline.

## Materials and Methods

We measured pH and concentration in 37 solutions from 4 different manufacturers and 13 different lots of commercially available solutions of 20 and 25% mannitol; 3, 5, 14.6, and 23.4% sodium chloride saline; and sterile water. Solutions were obtained by the Neuro ICU pharmacist through normal supply chain distribution. None of these samples or lots had been related to any FDA recalls. Proper storage and transportation was confirmed in accordance with usual clinical practices. Solutions were confirmed to be intact and unexpired, determined to be free from crystals or contaminants on visual inspection, and progressively labeled A through AK. All testing was conducted at a normal room temperature range of 20–25 degrees Celsius. All machines were calibrated and used in accordance with manufacturer specifications. All assays were performed at the University of Utah between September 2017 and February 2019.

pH was determined digitally (Orion 8103BNUWP Ultra pH probe with Orion 3-Star benchtop meter, Thermo Fisher Scientific Inc, West Valley City, Utah) and verified with litmus paper (pHydrion, Micro Essential Laboratory, Brooklyn, New York). Two investigators recorded measurements independently. Osmolality was determined by freezing point osmometry (model 3320, Advanced Instruments, Inc., Norwood, MA) and verified by vapor pressure osmometry (Vapro, ELITechGroup, Logan, Utah). Salinity/specific gravity was determined using portable refractometry (ETvalley, Shenzhen, China).

To visualize particulate matter, we pushed 1 mL of solutions A through V through 0.8-micron filter paper (Merck Millipore, Billerica, Massachusetts) and examined the dried filter under light microscopy. We corroborated these results using nephelometry with dynamic light scattering instrumentation (DynaPro Plate Reader II, Wyatt Technology, Santa Barbara, CA). With this technique, a laser is passed through a sample at an angle, and the intensity of scattered light is measured to determine the size-distribution of dissolved particles.

For all tests, a minimum of three measurements were taken. A one-way ANOVA with Tukey's *post-hoc* test to compare means across multiple groups was conducted in SAS version 9.4. Linear regression was conducted using GraphPad to assess labeled concentration-dependent trends. Fisher's exact test was conducted to evaluate proportions. Alpha was taken as 0.05 for all tests.

## Results

Our ANOVA model incorporated solution contents (i.e., 14.6% saline), type (i.e., saline), manufacturer, lot, and labeled concentration. Calculated *p*-value for this model was <0.0001. Sample characteristics and results are summarized in Table 1.

**Table 1:** Measured solution physical properties.

**ID**	**Contents**	**Type**	**Maker**	**Lot**	**Digital pH**	**Fp osmolality**	**Bp osmolality**	**Salinity/sg (%)**
A	Sterile water	Control	A	1	5.23	0 (0)	4.6 (0)	0 (0)
B	5% saline	Saline	A	2	5.26	1,597 (1,548–1,712)	1,576 (1,548–1,712)	4.65 (4.75–5.25)
C	5% saline	Saline	A	2	5.23	1,607 (1,548–1,712)	1576.8 (1,548–1,712)	4.65 (4.75–5.25)
D	5% saline	Saline	A	2	5.64	1,616 (1,548–1,712)	1570.8 (1,548–1,712)	4.6 (4.75–5.25)
E	20% mannitol	Mannitol	B	3	5.82	1,367 (869–961)	1287.4 (869–961)	15.6 (19–21)
F	20% mannitol	Mannitol	B	3	6.32	1,345 (869–961)	1285.2 (869–961)	16 (19–21)
G	20% mannitol	Mannitol	B	3	6.51	1,407 (869–961)	1281.2 (869–961)	15.8 (19–21)
H	3% saline	Saline	A	4	5.21	950 (947–1,047)	938.8 (947–1,047)	2.7 (2.85–3.15)
I	3% saline	Saline	A	4	5.38	955 (947–1,047)	937.6 (947–1,047)	2.7 (2.85–3.15)
J	3% saline	Saline	A	4	5.39	946 (947–1,047)	937.6 (947–1,047)	2.75 (2.85–3.15)
K	20% mannitol	Mannitol	B	5	6.65	1,347 (869–961)	1,278 (869–961)	15.5 (19–21)
L	20% mannitol	Mannitol	B	5	6.80	1,356 (869–961)	1258.6 (869–961)	15.8 (19–21)
M	20% mannitol	Mannitol	B	5	6.64	1,334 (869–961)	1,278 (869–961)	16 (19–21)
N	23.4% saline	Saline	C	6	5.56	7,150 (6,164–6,814)	7483.2 (6,164–6,814)	21.6 (22.2–24.6)
O	23.4% saline	Saline	C	6	5.68	5,914 (6,164–6,814)	7482.4 (6,164–6,814)	21.6 (22.2–24.6)
P	23.4% saline	Saline	C	6	5.68	8,022 (6,164–6,814)	7487.2 (6,164–6,814)	21.4 (22.2–24.6)
Q	14.6% saline	Saline	B	7	5.85	4,880 (4,144–4,582)	4553.2 (4,144–4,582)	13.5 (13.8–15.3)
R	14.6% saline	Saline	B	7	5.75	4,387 (4,144–4,582)	4,516 (4,144–4,582)	13.4 (13.8–15.3)
S	14.6% saline	Saline	B	7	5.74	5,979 (4,144–4,582)	4616.4 (4,144–4,582)	15.7 (13.8–15.3)
T	3% saline	Saline	A	8	5.65	961 (947–1,047)	934.2 (947–1,047)	2.7 (2.85–3.15)
U	3% saline	Saline	A	8	5.42	960 (947–1,047)	936.4 (947–1,047)	2.7 (2.85–3.15)
V	3% saline	Saline	A	8	5.52	960 (947–1,047)	935.8 (947–1,047)	2.7 (2.85–3.15)
W	3% saline	Saline	A	9	5.01	944 (947–1,047)	944 (947–1,047)	2.8 (2.85–3.15)
X	3% saline	Saline	A	9	5.30	943.7 (947–1,047)	944 (947–1,047)	2.85 (2.85–3.15)
Y	3% saline	Saline	A	9	5.19	940.7 (947–1,047)	943 (947–1,047)	2.85 (2.85–3.15)
Z	3% saline	Saline	D	10	4.40	942 (947–1,047)	944 (947–1,047)	2.9 (2.85–3.15)
AA	3% saline	Saline	D	10	4.39	960.7 (947–1,047)	941 (947–1,047)	2.9 (2.85–3.15)
AB	3% saline	Saline	D	10	4.37	944 (947–1,047)	940 (947–1,047)	2.9 (2.85–3.15)
AC	25% mannitol	Mannitol	B	11	5.81	1475.7 (1,043–1,153)	1,422 (1,043–1,153)	20.1 (23.7–26.3)
AD	25% mannitol	Mannitol	B	11	4.57	1381 (1,043–1,153)	1,428 (1,043–1,153)	21 (23.7–26.3)
AE	25% mannitol	Mannitol	B	11	5.01	1,374 (1,043–1,153)	1,416 (1,043–1,153)	22.2 (23.7–26.3)
AF	23.4% saline	Saline	C	12	4.13	7,502 (6,164–6,814)	7,374 (6,164–6,814)	23.1 (22.2–24.6)
AG	23.4% saline	Saline	C	12	4.30	7,492 (6,164–6,814)	7,386 (6,164–6,814)	23.4 (22.2–24.6)
AH	23.4% saline	Saline	C	12	3.71	7,514 (6,164–6,814)	7,380 (6,164–6,814)	23.1 (22.2–24.6)
AI	20% mannitol	Mannitol	B	13	4.60	1,349 (869–961)	1,140 (869–961)	18 (19–21)
AJ	20% mannitol	Mannitol	B	13	4.51	1,308 (869–961)	1,146 (869–961)	16.8 (19–21)
AK	20% mannitol	Mannitol	B	13	4.51	1,317 (869–961)	1,170 (869–961)	18 (19–21)

*Industry standard pH for all solutions is 4-5-7.0. Industry standards for osmolality and salinity/specific gravity are labeled concentration ±5%. These are specified in parentheses following the measured parameter. ID, solution identifier; fp, freezing point; vp, vapor pressure; sg, specific gravity*.

**Figure 1:**
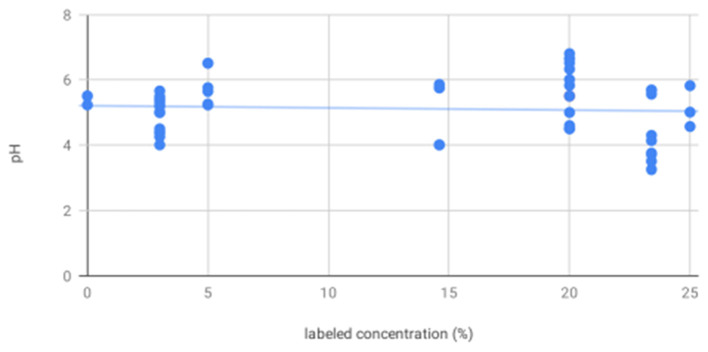
There is no association between labeled solution concentration and pH—scatter plot showing pH as measured by digital pH meter and litmus paper for all solutions shows no significant trend in pH as a function of increasing labeled solution concentration.

pH of all solutions was below physiological range (digital range 4.13–6.80). For digital pH measurements, mannitol (mean 5.65, standard deviation 0.94) was significantly less acidic than hypertonic saline (5.16, 0.60). 20% mannitol (5.82, 1.00) was less acidic than all other solutions, including 25% mannitol (5.13, 0.63), 3% (5.10, 0.46), 5% (5.38, 0.23), 14.6% (5.78, 0.06), and 23.4% (4.84, 0.89) saline, and sterile water (mean 5.23). Litmus testing results were concordant with digital pH results. Although all solutions were more acidic than physiological pH, there was no apparent correlation between pH and the specified solution concentration (*R*^2^ = 0.005, *p* = 0.60, Figure 1).

14/59 (24%) pH measurements conducted fell outside of manufacturer standards (4.5–7.0, per package inserts). In all 14/14 instances, measured pH was below 4.5. 0/18 mannitol pH measurements were outside of manufacturer range vs. 14/39 (36%) hypertonic saline, *p* = 0.0025. 2/23 (9%) pH measurements for manufacturer A were out of standards vs. 3/24 (13%) for B, 6/9 (67%) for C, and 3/3 for D, *p* < 0.0001.

**Figure 2:**
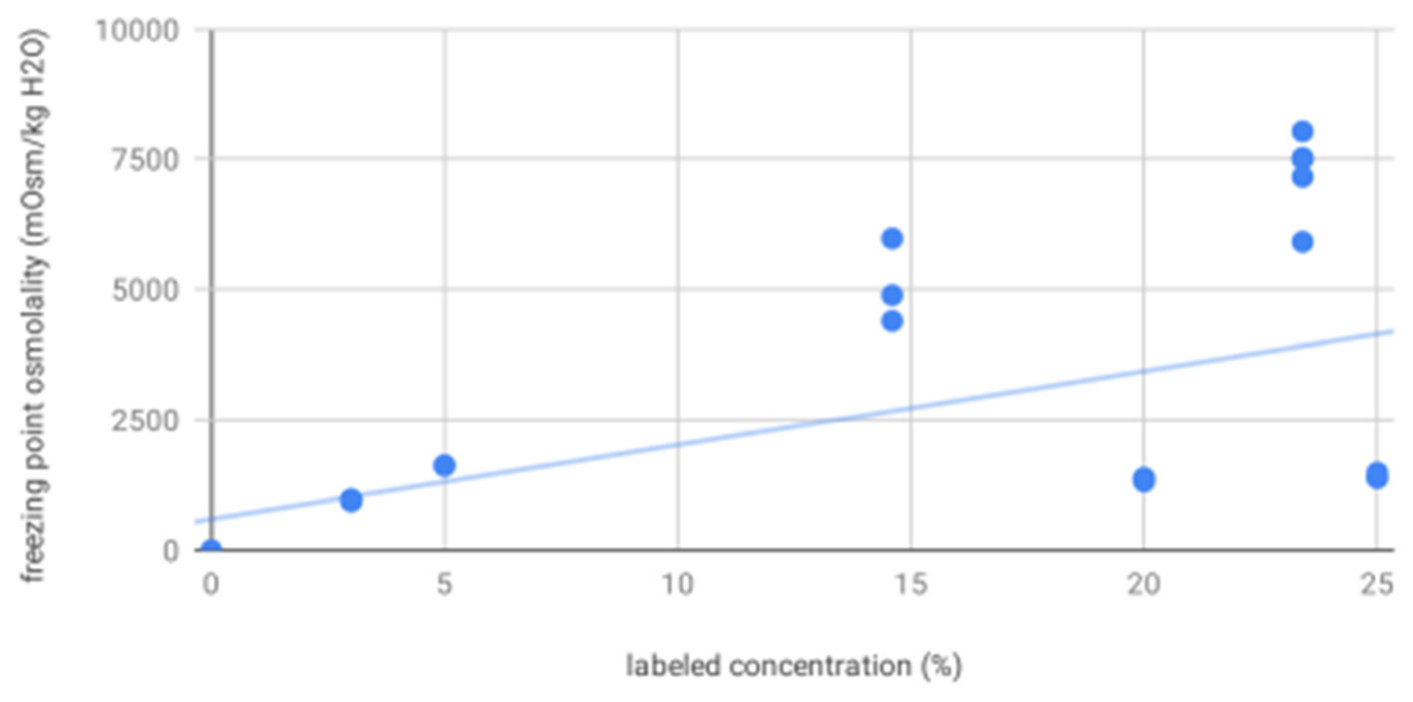
Freezing point osmolality increases with increasing labeled solution concentration—scatter plot showing osmolality as measured by freezing point depression for all solutions increases as a function of increasing labeled solution concentration, as expected.

For osmolality as measured by freezing point depression, 20% mannitol (1349.22, 22.86) was significantly less concentrated than 14.6% (5082.00, 815.00) and 23.4% (7265.67, 718.49) saline; 25% mannitol (1410.33, 56.98), 3% saline (952.17, 8.33), and 5% saline (1613.00, 5.57) were less concentrated than 14.6 and 23.4% saline. As expected, osmolality as measured by freezing point depression increased with increasing labeled concentration (*R*^2^ = 0.29, *p* = 0.0006, Figure 2).

**Figure 3:**
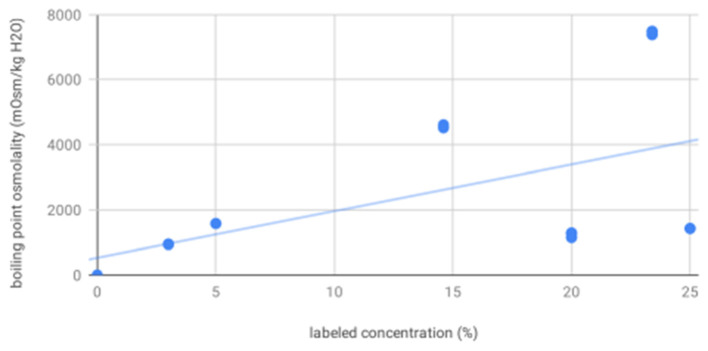
Boiling point osmolality increases with increasing labeled solution concentration—scatter plot showing osmolality as measured by vapor point elevation for all solutions increases as a function of increasing labeled solution concentration, as expected.

For osmolality as measured by vapor point elevation, all solutions were significantly different from each other. These were, from low to high concentration: sterile water (4.60), 3% saline (939.70, 3.49), 20% mannitol (1236.04, 64.05), 25% mannitol (1422.00, 6.00), 5% (1574.53, 3.26), 14.6% (4561.87, 50.76), and 23.4% saline (7432.13, 57.26). As expected, osmolality as measured by vapor point elevation increased with increasing labeled concentration (*R*^2^ = 0.29, *p* = 0.0005, Figure 3). These vapor point elevation results were concordant with osmolality as measured by freezing point depression.

**Figure 4:**
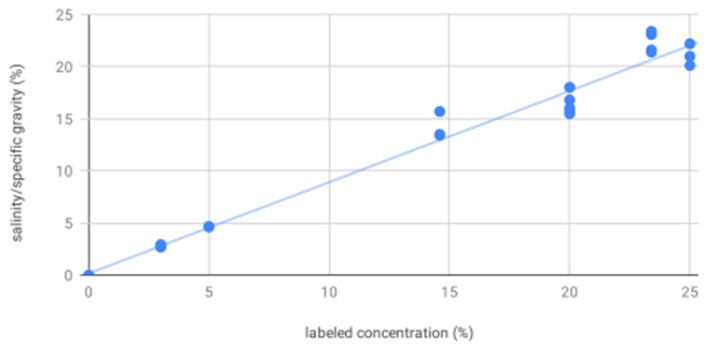
Salinity/specific gravity increases with increasing labeled solution concentration—scatter plot showing salinity/specific gravity as measured by portable refractometer for all solutions increases as a function of increasing labeled solution concentration, as expected. These results showed measured values consistently and proportionally less than labeled concentration by a weighted mean difference of 18% for mannitol and 6% for hypertonic saline. This may be a result of crystallization. As with freezing point depression and vapor point elevation, there was increased variability at higher concentrations.

For salinity/specific gravity as measured by portable refractometry, all solutions were significantly different from each other, including: 20% (16.39, 0.99) and 25% (21.10, 1.05) mannitol; 3% (2.79, 0.09), 5% (4.63, 0.03), 14.6% (14.20, 1.30), and 23.4% (22.37, 0.92) hypertonic saline; and sterile water (0.00). As expected, salinity/specific gravity as measured by portable refractometry increased with increasing labeled concentration (*R*^2^ = 0.98, *p* < 0.0001, Figure 4).

Measured concentration—including both osmometry and refractometry—differed significantly from labeled concentration, and there was a great deal of variability with measurements. 85/111 (77%) total concentration measurements fell out of manufacturer standards (published parameter ±5%). All 36/36 mannitol concentration measurements were out of standards vs. 48/72 (67%) for hypertonic saline, *p* < 0.0001. All mannitol specific gravity measurements were below the standard range and all mannitol osmolality measurements were above the standard range, which may suggest solvent evaporation or presence of impurities, intermediates, or breakdown products. 23/39 (59%) concentration measurements for manufacturer A were out of standards vs. 42/45 (93%) for B, 15/18 (83%) for C, and 5/9 (56%) for D, *p* = 0.0004.

4/22 (18%) comparisons between solutions with the same contents from the same manufacturer from different lots were statistically significantly different, whereas 3/12 (25%) comparisons between solutions with the same contents from different manufacturers were statistically significantly different; the statistical comparison between these proportions was not statistically significantly different, *p* = 0.68. These results may be underpowered given our small sample size, or they may suggest our findings are independent of manufacturer and lot.

**Figure 5:**
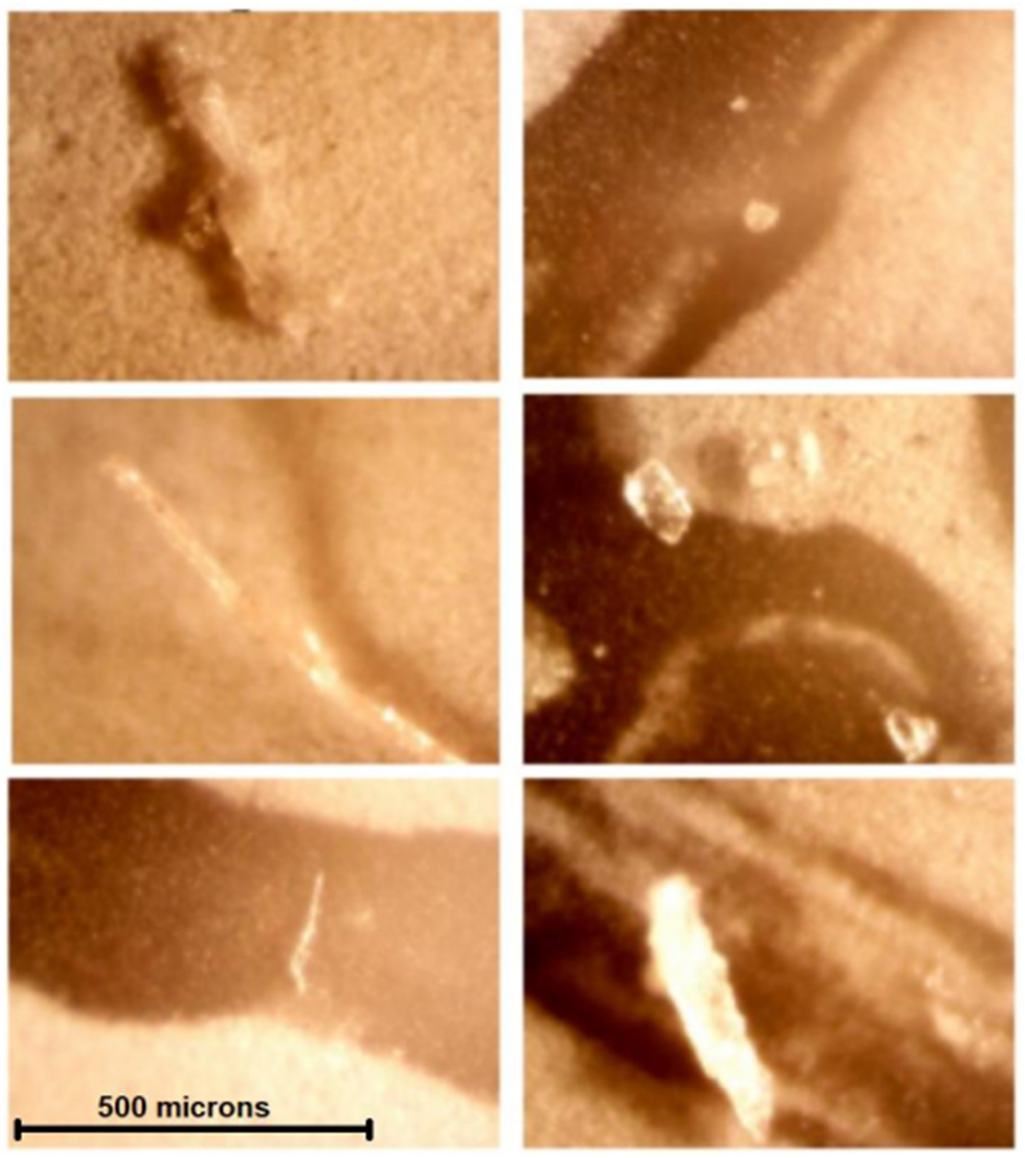
Contaminants or crystals were found in all solutions examined by light microscopy—All images were obtained at the same magnification. Noncrystalline contaminants or crystals visualized are considerably larger than human capillary beds (5–10 microns). These are, clockwise, from top left, solutions A (sterile water), B (5% saline), I (3% saline), G (20% mannitol), L (20% mannitol), and P (23.4% saline).

All solutions examined on light microscopy—including sterile water, mannitol, and hypertonic saline solutions—were found to contain crystalline and/or non-crystalline particulate matter up to several hundred microns in diameter (Figure 5). From nephelometry, particulate matter was detected in 20/22 (91%) solutions, with mean particle diameter ranging from 0.1 to 96.3 microns. Based on standard classification used in this technique, 6/20 (30%) particulate matter containing solutions were highly monodisperse (homogeneous mixtures); 12/20 (60%) solutions were monodisperse and 2/20 (10%) solutions were polydisperse (heterogeneous mixtures). Nephelometry results are presented in further detail in Table 2.

**Table 2:** Dynamic light scattering nephelometry data for solutions A through V.

**ID**	**Contents**	**Mean particle diameter (μm)**	**% Polydispersity**	**Polydispersity index**
A	Sterile water	0	0	0
B	5% NaCl	45.6	2.02	0.02
C	5% NaCl	83.9	15.32	0.15
D	5% NaCl	35.3	13.74	0.14
E	20% mannitol	43.0	22.11	0.22
F	20% mannitol	55.2	8.75	0.09
G	20% mannitol	0.1	8.27	0.08
H	3% NaCl	0.1	0.00	0.00
I	3% NaCl	0	0.00	0.00
J	3% NaCl	0.068	3.99	0.04
K	20% mannitol	20.2	0.00	0.00
L	20% mannitol	0.6	9.49	0.09
M	20% mannitol	0.2	12.15	0.12
N	23.4% NaCl	0.1	6.60	0.07
O	23.4% NaCl	0.1	3.59	0.04
P	23.4% NaCl	0.3	18.21	0.18
Q	14.6% NaCl	75.7	9.41	0.09
R	14.6% NaCl	12.2	17.49	0.17
S	14.6% NaCl	43.6	13.94	0.14
T	3% NaCl	96.3	10.08	0.10
U	3% NaCl	57.7	6.36	0.06
V	3% NaCl	0.6	22.96	0.23

*Here we include a measure of central tendency as mean particle size and a measure of variance as polydispersity. Polydispersity describes the width of the Gaussian distribution. The percent polydispersity may be used to broadly characterize a solution as monodisperse (<20%) or polydisperse (≥20%). The polydispersity index is a dimensionless and scaled number that is calculated from correlated data. It has been used to indicate the fitness of the sample for analysis by dynamic light scattering nephelometry. Solution samples with polydispersity indices between 0.05 and 0.7 are typically good candidates for DLS analysis. Values > 0.7 indicate a broad diversity of particle size ranges. Mean particle sizes varied widely between the solutions, ranging from 0 μm (solutions A and I) to 96.3 μm (solution T). In accordance with their percent polydispersity, solutions E (22.11%) and V (22.96%) would be characterized as polydisperse; all other solutions are monodisperse. Solutions B, H, I, J, K, and O, have a polydispersity index below 0.05. Values <0.05 are rarely seen and usually indicate highly monodisperse standard solutions. Nevertheless, the average particle sizes in these 6 highly monodisperse solutions range from 0 to 45.6 μm. It is possible that because many of these particle sizes are in the micron range rather than nano range, the machine may have had some difficulty in analysis*.

## Discussion

Though mannitol has long-been the workhorse hyperosmolar therapy, a growing body of literature suggests that hypertonic saline is also effective in treating elevated intracranial pressure from a variety of causes (12). Although it has been suggested that hypertonic saline may be superior to mannitol in terms of rate, duration, effect size, and side effect profile, controversy remains (13–19). It may be reasonably inferred that hypertonic saline has the same osmotic effect as mannitol (20–22), yet crucial key differences may exist for additional mechanisms of action that have yet to be fully explored, and there may be particular circumstances or indications that favor one agent over the other (23–30).

Through our investigations of pH, osmolality, and salinity/specific gravity, we were able to detect many significant differences in physical properties among commercially available mannitol and hypertonic saline solutions taken from our ICU that may inform the decision of which hyperosmolar therapy to use for a particular patient. In summary, we found the following:

### pH of all Hyperosmolar Solutions Was Considerably Below Physiological Range

We detected pH values considerably below physiological range (7.35–7.45)—and manufacturer standards (4.5–7.0)—across multiple solution contents, types, manufacturers, lots, and labeled concentrations, using multiple measurement modalities. The effect of hyperosmolar infusion on acid/base homeostasis is poorly understood. The differences we detected may be relevant, especially for acidotic patients. More research is needed on this matter, including correlation of measured solution properties with clinical and laboratory outcomes.

### Mannitol Was Less Acidic Than Hypertonic Saline

Our results showed mannitol was less acidic than hypertonic saline. Previous investigations have shown the development of hyperchloremic metabolic acidosis after large saline infusions (31–34). This has been shown to be an independent risk factor for mortality in critically ill patients (35–37). Among studies of neurocritical care patients in particular, Riha et al. observed increased in-hospital mortality among patients with moderate hyperchloremia during 3% hypertonic saline infusion (38). Sadan et al. showed a strong association between hyperchloremia and acute kidney injury and between acute kidney injury and mortality in subarachnoid hemorrhage patients (39). Finally, Huang et al. demonstrated that hyperchloremia and increasing serum chloride were associated with increased odds of 30-day mortality and poor outcome after 6-months in a population of critically ill stroke patients (40). While none of these studies demonstrates a causal relationship between hypertonic saline infusion and poor clinical outcomes in neurocritical care patients, it may be preferable to choose mannitol over hypertonic saline in patients with concerning acidosis who require hyperosmolar therapy. In any case, one should be conscious of the propensity for such patients to develop hyperchloremic metabolic acidosis and the effects this can have on clinical outcomes and monitor accordingly. Our results suggest that further direct investigation of this question is warranted.

### Measured Concentration Differed From Labeled Concentrations

Osmolality and salinity/specific gravity measurements were inconsistent with labeled concentrations across multiple solution contents, types, manufacturers, and lots using multiple measurement modalities. This suggests the presence of occult crystallization, evaporation, or impurities, intermediates, or breakdown products in solution. More research should be undertaken to investigate how variance in hyperosmolar solution contents may explain variant clinical responses and outcomes. In the meantime, our results suggest that clinicians may want to be cautious in administering the minimum effective dose of hyperosmolar therapy in conjunction with real-time monitoring for treatment effects and adverse effects such as those that may be caused by over- or under-diuresis or end-organ damage.

### Mannitol Was More Likely to Be Out of the Specified Concentration Range Compared to Hypertonic Saline

Mannitol may be supersaturated at room temperature, and manufacturer labels state that it should be warmed before use if crystallization has occurred. We were unable to find any such recommendation for hypertonic saline, as the concentration at which aqueous sodium chloride becomes supersaturated is above that of the solutions we tested. Our tests were all conducted at room temperature, and no crystals were identified on gross visual inspection. If crystallization occurred, our results may suggest a need to warm solutions even without gross evidence of crystallization. Our results add to the existing literature demonstrating an inconsistent dose-response relationship between hyperosmolar solutions and intracranial pressure (41–43). While we cannot comment specifically on results of past studies or infer any effect on intracranial pressure from our data, our results suggest that future investigations of the dose-response relationship between hyperosmolar solutions and intracranial pressure may wish to consider validating their own findings by directly measuring the physical properties of the solutions given.

### Large Particulate Matter Was Found in all Solutions we Examined

All solutions were found to contain particulate matter on both light microscopy and nephelometry. Nephelometry is an analytical chemistry method to measure intensity of scattered light and extrapolate particle size distribution within a solution. Nevertheless, this technique is limited to size analysis and does not discern specific chemical identity. We were therefore unable to definitively determine if particulate matter in our samples was due to crystallization, contamination, or both. Although speculative, the presence of non-degradable contaminants such as microplastics, which are now ubiquitous in the environment (44), in intravenous fluids would be a concerning finding warranting aggressive additional investigation.

Many solutions contained particles considerably larger than human capillary beds (5–10 μm). Such particulate matter in the bloodstream may be thrombogenic or obstruct blood vessels (45–47), thereby depriving the tissues of oxygen or other nutrients or impeding the expeditious diuresis that is the very purpose of hyperosmolar therapy. Furthermore, direct injury to body tissues including the lung, kidney, and brain may result from crystal or contaminant deposition, which may be compounded by a patent foramen ovale. It should be noted that intravenous fluids undergo sterile processing and are not filtered during manufacturing.

### Study Limitations

There are many studies that examine clinical outcomes or surrogate endpoints (such as effect on intracranial pressure) in patients undergoing mannitol or hypertonic saline therapy. Likewise, there are many basic science studies involving the administration of hyperosmolar solutions under idealized laboratory conditions that rely on precise compounding and controlled experiment. We believe this is the first study that interrogates the physical properties of commercially-available hyperosmolar solutions as they actually exist in the ICU—that is to say, what actually gets infused into patients. While these solutions are manufactured and tested under tightly regulated systems of quality control, we found that, despite a remarkably wide range of values permitted, a large number of solutions fell out of that wide range when subjected to our own rigorous testing. We were able to corroborate our results using multiple complementary techniques—i.e., both digital and litmus paper measurement of pH; precise freezing point depression and boiling point elevation methods for determining osmolality along with an analog technique for determining salinity/specific gravity. Our divergent results do not speak directly to whether manufacturer standards and quality control are flawed, nor can we attribute causality to storage or transportation factors. Nevertheless, the high degree of variance in pH and concentration, along with evidence of widespread particulate matter, that we found in hyperosmolar solutions obtained through our normal supply chain distribution is concerning and should prompt additional investigation.

Our study also has several weaknesses: since our study is a novel characterization, we were not certain what we would find. Although the 37 solutions from 4 manufacturers and 13 lots that we did evaluate represents the widest range we were able to procure over a period of several months, perhaps due to supply chain difficulties, overall we did not investigate a large number of solutions, and our results were likely affected by this small sample size. More solutions from different manufacturers and lots must be sampled to more accurately characterize manufacturer- and lot-specific central tendency and variance. Additionally, we conducted our experiments at room temperature, rather than at body temperature. As mentioned above, mannitol may be supersaturated at room temperature, and manufacturer labels state that it should be warmed before use if crystallization has occurred. Nevertheless, no crystals were identified on gross visual inspection. Hyperosmolar solutions are often used emergently, and it is our experience that mannitol solutions are seldom warmed before use if there is no gross evidence of crystallization. It was our aim to recapitulate as faithfully as possible the conditions under which these solutions are actually administered. One would expect the solutions to warm in the body, but the rapidity and extent of crystals dissolving is uncertain in this circumstance. It would have been a useful investigation to warm the solutions to body temperature before passing them through filters or interrogating them using nephelometry with dynamic light scattering instrumentation in order to determine the effect of temperature on our detection of particulate matter. Furthermore, there are no recommendations for warming hypertonic saline; nevertheless, particulate matter was ubiquitous in all solutions and solution types. This warrants future investigation. Likewise, while solution package inserts allow for buffering to ensure the recommended pH range, it is our experience that pH of solutions is seldom checked before infusion, particularly in the emergent situations that may call for mannitol or hypertonic saline. If the patient experiences an acidosis following hyperosmolar therapy, it may be attributed to underlying disease rather than the possibility that it may be iatrogenic. As stipulated above, our findings confirm that clinicians should be particularly mindful if a patient develops a hyperchloremic metabolic acidosis following hypertonic saline infusion. Finally, our study is hypothesis generating: it is basic science conducted in a laboratory and cannot be directly extrapolated to patient care. Additional clinical or pathological research involving patients—i.e., causal investigations involving acidosis and alkalosis or deposition of crystal or non-crystalline contaminants in tissues following hyperosmolar therapy—is necessary to establish the clinical relevance of our findings.

## Conclusion

In conclusion, our purpose was to present a novel and practical characterization of commercially available hyperosmolar solutions used for critically ill patients—including various concentrations of mannitol and hypertonic saline. Even with the remarkably broad range permitted for the physical properties of hyperosmolar solutions, values for many solutions fell outside of these permitted ranges. We found that pH of all hyperosmolar solutions was considerably below physiological range, although mannitol was less acidic than hypertonic saline. This finding may be relevant for patients with acid/base disturbances. Future clinical studies should investigate the development of acidosis following treatment with either agent. Measured solution concentrations differed considerably from labeled concentrations, suggesting occult crystallization or non-uniformity and potentially reduced effectiveness or potential for harm; mannitol was more likely to be outside of concentration standards compared to hypertonic saline. Crystalline and non-crystalline particulate matter was also observed in all solutions, even those that are not known to crystallize at room or physiological temperatures. Further research is needed to characterize manufacturer and lot effects and to determine if particulate matter in these solutions can induce thrombogenesis or occlude the microvasculature.

## Data Availability Statement

The raw data supporting the conclusions of this article will be made available by the authors upon request.

## Author Contributions

CCa: conceptualization, data curation, formal analysis, investigation, methodology, project administration, software, writing—original draft, and visualization. JS: conceptualization, data curation, formal analysis, investigation, methodology, project administration, software, and writing—original draft. JR: conceptualization, data curation, formal analysis, investigation, methodlogy, project administration, resources, software, supervision, and writing—original draft. CCo, GD, CF, LK, and KM: methodology, resources, and writing—reviewing and editing. ER: methodology, project administration, resources, and writing—reviewing and editing. BS: writing—reviewing and editing. GH: conceptualization, funding acquisition, methodology, project administration, resources, supervision, and writing—review and editing. All authors contributed to the article and approved the submitted version.

## Conflict of Interest

The authors declare that the research was conducted in the absence of any commercial or financial relationships that could be construed as a potential conflict of interest.
